# Efficiency and safety of desloratadine in combination with compound glycyrrhizin in the treatment of chronic urticaria: a meta-analysis and systematic review of randomised controlled trials

**DOI:** 10.1080/13880209.2021.1973039

**Published:** 2021-09-13

**Authors:** Yulong Wen, Yidan Tang, Miaoyue Li, Yu Lai

**Affiliations:** a School of Basic Medicine, Chengdu University of Traditional Chinese Medicine, Chengdu, China; b School of Pharmacy, Chengdu University of Traditional Chinese Medicine, Chengdu, China

**Keywords:** Immune regulation, loratadine

## Abstract

**Context:**

Desloratadine, an H1 receptor antagonist, is suggested as an effective first-line drug for chronic urticarial (CU). However, the efficacy of desloratadine alone is limited, and the recurrence rate of CU is relatively high.

**Objective:**

We sought to evaluate the efficacy and clinical feasibility of desloratadine in combination with compound glycyrrhizin in the treatment of CU.

**Materials and methods:**

A systematic literature search was conducted in the databases of the China National Knowledge Infrastructure Database, VIP, WanFang, PubMed, and Web of Science using subject terms: “Chronic urticaria”, “Loratadine”, and “Compound glycyrrhizin”. Randomised controlled trials (RCTs) that compared the efficiency and safety of the combination treatment with desloratadine alone starting from January 1, 2014 until February 10, 2021 were selected by two co-first authors independently, and the extracted data were analysed using Rev Man 5.3 software.

**Results:**

Fourteen RCTs were included in our meta-analysis with a total of 1501 patients. The results showed that the combination treatment yielded a better treatment effect (total response rate: RR = 1.23, 95% CI: 1.17 to 1.29, *p* < 0.00001; cure rate: RR = 1.50, 95% CI: 1.30 to 1.73, *p* < 0.00001), lower recurrence rate as well as superior immune improvement than the treatment with desloratadine alone. In addition, there was no significant difference in the safety of the two treatments.

**Discussion and Conclusion:**

The combination of desloratadine and compound glycyrrhizin is a promising treatment for CU and is associated with decreased serum IgE level and improved proportions of CD4+ T and CD8+ T cells.

## Introduction

Chronic urticaria (CU) is a type I hypersensitivity reaction that is caused by a variety of complicated factors. The course of this disease usually lasts for 6 weeks or more. It has been reported that more than 5% of the general population is affected by CU, and it is more common in adults (Mastrorilli et al. 2019). The main symptom of the patients in the clinic is recurrent wheals accompanied by severe itching. Additionally, patients often experience more severe symptoms in the evening, which seriously affects their daily life and sleep quality (Ma 2014; Hu 2016). However, there is currently no cure for this disease, and its pathogenesis is still not entirely clear at present (Yuan 2018).

According to most of the current hypotheses, CU is caused by the action of histamines and the H receptor involving the pathogenesis of autoimmunity (Su et al. 2020). Therefore, antihistamine drugs are widely applied in clinical treatment. Desloratadine, a second-generation H1 receptor antagonist, is highly selective for peripheral H1 receptors (Liang et al. 2014). In a related study, it was suggested that the efficacy and safety of desloratadine in the treatment of CU is obviously superior to that of other antihistamines, such as cetirizine, mizolastine, or loratadine. As a result, it is considered to have good efficacy and reliable safety in the treatment of urticaria (Lang et al. 2019). Nevertheless, the efficacy of desloratadine alone is limited, and the CU recurrence rate is relatively high (Maurer et al. 2013; Sui et al. 2018).

Compound glycyrrhizin is an immune modulator comprised of glycyrrhizin, l-cysteine hydrochloride and glycine (Duan 2018). Its aglycone is relatively similar to the structure of corticotrophin releasing hormone (CRH), which can promote the activity of the adrenal corticosteroids, achieving anti-inflammatory and anti-allergic effects (Cui et al. 2017). Furthermore, as shown in clinical trials, it has no severe adverse reactions compared to CRH (Wu 2018). In addition, compound glycyrrhizin can reduce the level of IgE, increase the cellular function of Th1 cells and inhibit the activity of Th2 cells. As a result, compound glycyrrhizin can prevent and treat urticaria, and it has a good effect (Cui et al. 2017; Duan 2018; Wu 2018). According to the particular efficiency of compound glycyrrhizin as an immune modulator and its significant safety compared to CRH among plenty of Randomised controlled trials (RCTs) in treating CU, we selected it as another drug in the combination treatment in this study.

Over the past few years, a combination of desloratadine and compound glycyrrhizin has been recommended in treating CU. RCTs of the two drugs applied in combination have been conducted (Li et al. 2015; Zhang 2015; Sun 2016; Fan 2017; Qian 2017; Ying and Shi 2017; Zang 2017; Zeng et al. 2017; Duan 2018; Hang 2018; Sheng et al. 2018; Deng 2019; Gou 2019; Peng 2019). An ideal therapeutic effect on CU after applying this combined treatment has been shown in clinical trials. However, such experimental results are not objective or comprehensive enough, and most of these RCTs failed to explain the cause of this combination therapy from the perspective of a specific pathogenesis.

To comprehensively and objectively assess the clinical efficacy and safety of this combined treatment, we performed this meta-analysis. We also expect that this analysis can assist other researchers in finding out more about the application of antihistamines combined with immune modulators in treating CU to identify more ideal treatments for CU in the future.

## Materials and methods

### Search strategy

A systematic literature search was conducted in Chinese and English databases among the China National Knowledge Infrastructure Database (CNKI), VIP, WanFang, PubMed and Web of Science. The retrieval strategy was set up using the following subject terms: “Chronic urticaria”, “Loratadine”, and “Compound glycyrrhizin” were combined as Chinese search terms, and “Urticaria”, “Compound glycyrrhizin”, and “Desloratadine” were combined as English search terms. All relevant studies considered in this meta-analysis were published from January 1, 2014 until February 10, 2021.

### Eligibility criteria

#### Inclusion criteria

1. Randomised controlled studies on patients with CU treated with a combination of desloratadine and compound glycyrrhizin;
2. The patients included in the studies were diagnosed with CU with at least a 6-week course;
3. The participants did not take any antihistamine or immune modulator recently;
4. The age of the patients included in the studies was above 16.

#### Exclusion criteria

1. Studies about urticaria but mixed with other illnesses;
2. Animal experiments, studies with nebulous or incomplete data on outcome indicators;
3. Over 1 month on the cycle of treatment. Most related articles limited the treatment course to 1 month or 4 weeks.

### Interventions

The included randomised trials in this systematic review used the following design: the experimental group was treated with a combination of desloratadine and compound glycyrrhizin. The control group was treated only with desloratadine. In both groups, the cycle of treatment was 1 month without any restriction of the treatment dose. In addition, there was no limit on blinding methods or the sex, race or geographical region of the participants.

### Outcome definitions

(1) The total response rate was mostly evaluated based on clinical symptom changes in the patients. The clinical symptoms of the two groups before and after treatment, including the number of wheals, the diameter of the wheals, the degree of itching and the duration of the wheals, were assessed by a 4-grade scoring system. TSS (total symptom score) = (total score before treatment − total score after treatment)/total score before treatment × 100%. (TSS ≥90% without relapse within half a year means cured. 60%≤TSS<90% means effective and TSS<60% means invalid). The total response rate = (number of cured cases + effective cases)/the total number of patients × 100%; (2) cure means the TSS of the patients was greater than 90% and there was no recurrence within half a year. The cure rate = number of cured cases/the total number of patients × 100%; (3) the recurrence rate was recorded with follow-up after half or one year; (4) adverse reactions were the main symptoms, including dry mouth, nausea, headache, dizziness, fatigue, and drowsiness; and (5) serum total IgE level, CD4+ and CD8+ were immune indicators.

### Data extraction

The final articles included in this analysis were screened by two reviewers independently. After excluding duplicated studies, the titles and abstracts of the remaining articles were reviewed. Then, the full texts of the remaining studies were scrutinised independently by two reviewers. If there were disagreements on the final included articles between the two reviewers, the dispute was resolved by a third evaluator. Then, the basic data were extracted, including the first author, year of publication, demographic characteristics (age and sex), specific intervention plans, characteristics of the experimental group and control group and the outcome data involving the above outcome indicators. The results of the data extraction were reviewed by another author.

### Quality assessment

According to the bias risk assessment recommended by Review Manager [Version 5.3], the evaluation criterion included: (1) whether random sequences were generated; (2) whether there was random allocation concealment; (3) whether the participants and personnel were blinded to the intervention; (4) whether the assessment of the results data was blinded; (5) whether the results data were integrated; (6) selective reporting of the study results; and (7) other forms of bias. The Cochrane Correspondence Network RCT evaluation tool was used to evaluate each item according to low risk (+), unknown risk (?), and high risk (−). The quality evaluation of the literatures was conducted by group discussions.

### Statistical analysis

Meta-analysis was carried out using RevMan5.3 software. In this study, the risk ratio (RR) and its 95% CI were used as the effect analysis statistics. The variations in CD4 and CD8 and the changes in serum total IgE levels were summarised using the mean difference (MD) with 95% CI. Statistical heterogeneity was assessed by applying the chi-square test, the degree of heterogeneity was identified using the I^2^ value, and an I^2^ of <50% indicated low heterogeneity. A fixed-effect model was used to analyse the outcomes with low heterogeneity. Otherwise, a random-effect model was used. The P value threshold for statistical significance was set at 0.05 for effect sizes. A funnel plot was used to test for publication bias.

## Results

### Search results

**Figure 1.**
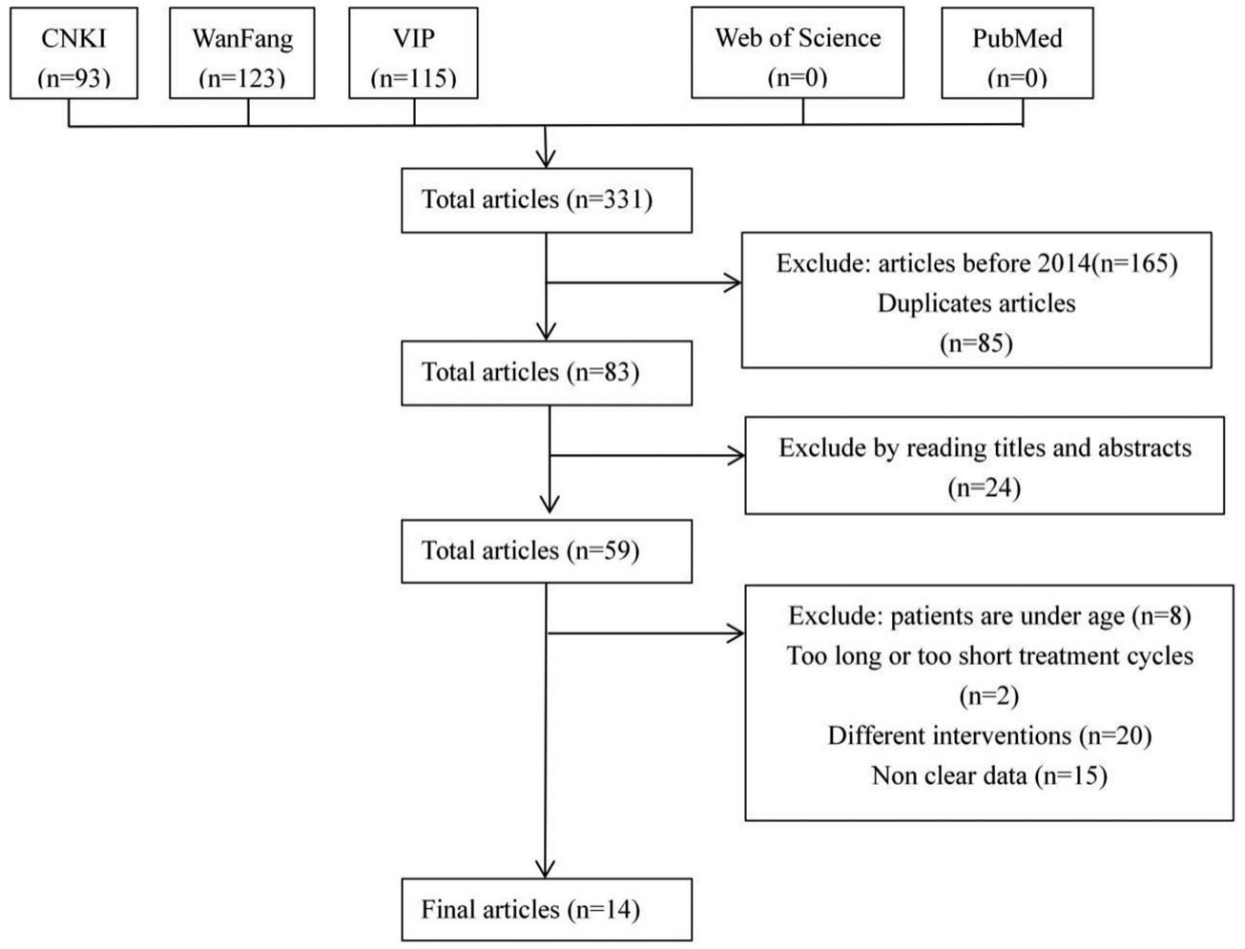
Study selection process for the meta-analysis.

According to the retrieval strategy, a total of 331 relevant studies were included in this analysis. Among them, none of the literatures came from PubMed or Web of Science, while there were 93 articles from CNKI, 123 from WanFang and 115 from VIP. After removing duplicated articles and studies before 2014, 83 articles were retained. Then, 69 articles were excluded because of failure to meet our inclusion criteria. Eventually, 14 eligible articles (Li et al. 2015; Zhang 2015; Sun 2016; Fan 2017; Qian 2017; Ying and Shi 2017; Zang 2017; Zeng et al. 2017; Duan 2018; Hang 2018; Sheng et al. 2018; Deng 2019; Gou 2019; Peng 2019) were included in our meta-analysis. The process and the results of the literature screening are shown in Figure 1.

### Research characteristics

The 14 included RCTs (Li et al. 2015; Zhang 2015; Sun 2016; Fan 2017; Qian 2017; Ying and Shi 2017; Zang 2017; Zeng et al. 2017; Duan 2018; Hang 2018; Sheng et al. 2018; Deng 2019; Gou 2019; Peng 2019) were published between 2014 and 2020 with a total of 1501 patients, including 755 in the experimental group and 746 in the control group. The patients were all Chinese, and their age was above 16. In addition, the course of disease ranged from 6 weeks to 10 years, and the course of treatment was limited to 1 month or 4 weeks. The specific information is shown in Table 1.

**Table 1.** The basic characteristics of the 14 RCTs.

Author/year	Groups	Sample size	Age (median or mean or range)	Sex (male/female)	Course of disease (mean or range)	Course of treatment	Intervention	Outcomes
Li et al., 2015	EP	70	19-57	31/39	3 months-2 years	1 month	A + C	abcefg
	CG	70	20-55	30/40	3 months-2 years	1 month	A	
Zhang, 2015	EP	46	Unknown	29/17	2 months-9 years	4 weeks	A + D	abg
	CG	38	Unknown	20/18	2 months-9 years	4 weeks	A	
Sun, 2016	EP	55	20-68	30/25	2 months-2 years	1 month	A + C	aef
	CG	55	18-65	28/27	1 months-2 years	1 month	A	
Ying and Shi, 2017	EP	40	22-52	25/15	Unknown	4 weeks	A + D	abcdefg
	CG	40	21-53	24/16	Unknown	4 weeks	A	
Fan, 2017	EP	45	18-66	24/21	40 days-15 months	4 weeks	A + C	abg
	CG	45	17-67	23/22	39 days-15 months	4 weeks	A	
Zeng et al., 2017	EP	90	18-74	44/46	3 months-4.4 years	4 weeks	B + C	bcdefg
	CG	90	18-72	45/45	3 months-4.3 years	4 weeks	B	
Zang, 2017	EP	55	17-60	Unknown	6 months-10 years	1 month	A + C	acg
	CG	55	17-60	Unknown	6 months-10 years	1 month	A	
Qian, 2017	EP	60	36.23 ± 6.75	30/30	(1.62 ± 0.38) years	1 month	A + C	acefg
	CG	60	36.94 ± 4.53	31/29	(1.82 ± 0.45) years	1 month	A	
Hang, 2018	EP	51	18-67	Unknown	2 months-2 years	4 weeks	A + D	abg
	CG	51	18-67	Unknown	2 months-2 years	4 weeks	A	
Duan, 2018	EP	63	36.7 ± 11.4	29/34	(14.6 ± 4.5) years	1 month	A + C	acg
	CG	62	35.8 ± 10.9	24/38	(15.1 ± 4.2) years	1 month	A	
Sheng et al., 2018	EP	35	18-64	15/20	2 months-3 years	4 weeks	A + E	cef
	CG	35	18-63	14/21	2 months-3 years	4 weeks	A	
Deng, 2019	EP	45	25-49	Unknown	1.8-7.6 years	30 days	A + C	acef
	CG	45	25-49	Unknown	1.8-7.6 years	30 days	A	
Gou, 2019	EP	41	27-66	16/25	3 months-6 years	4 weeks	A + E	ag
	CG	41	29-68	17/24	4 months-5 years	4 weeks	A	
Peng, 2019	EP	59	32 ± 2.09	22/37	6 weeks-33 months	4 weeks	A + D	abg
	CG	59	31 ± 2.12	28/31	6 weeks-34 months	4 weeks	A	

EP: experimental group; CG: control group; A. desloratadine: 5 mg po qd; B. desloratadine: 8.8 mg po qd; C. compound glycyrrhizin tablets:75 mg po tid; D. compound glycyrrhizin tablets: 50 mg po tid; E. compound glycyrrhizin capsule po tid; a. total effective rate; b. adverse reaction; c. recurrence rate; d. serum IgE level; e. CD4+; f. CD8+; g. cure rate.

### Summary of the quality and bias risk of the included trials

**Figure 2.**
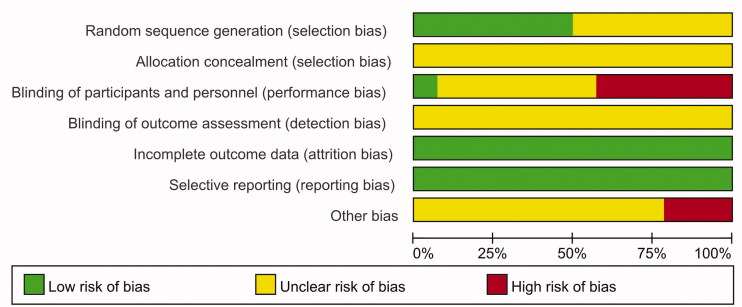
Risk of bias graph.

**Figure 3.**
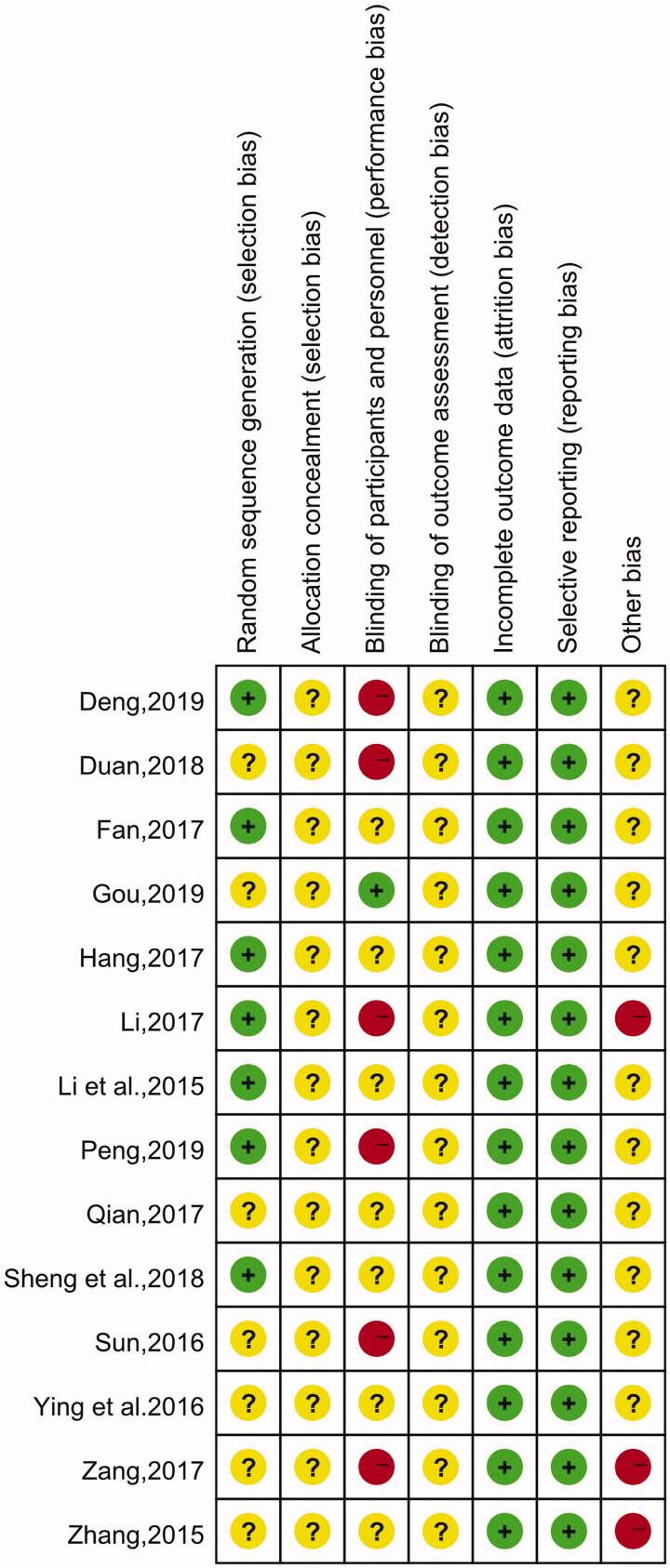
Risk of bias summary.

Among the 14 studies included, only 5 mentioned the application of a random number table (Li et al. 2015; Zeng et al. 2017; Hang 2018; Sheng et al. 2018; Deng 2019), and only 1 study described the blinding method (Gou 2019). The rest of the articles failed to describe a randomisation method, concealment of allocation, the blinding method, blinding of estimators, the absence of figures, or selection bias. The quality assessment results are shown in Figures 2 and 3.

### Outcome measures

Twelve studies reported the total response rate (Li et al. 2015; Zhang 2015; Sun 2016; Fan 2017; Qian 2017; Ying and Shi 2017; Zang 2017; Duan 2018; Hang 2018; Deng 2019; Gou 2019; Peng 2019), and some of the included articles reported other outcome measures. Among the 14 articles, 11 recorded the cure rate (Li et al. 2015; Zhang 2015; Fan 2017; Qian 2017; Ying and Shi 2017; Zang 2017; Zeng et al. 2017; Duan 2018; Hang 2018; Gou 2019; Peng 2019), the recurrence rate was described in 7 studies (Li et al. 2015; Qian 2017; Ying and Shi 2017; Zang 2017; Duan 2018; Sheng et al. 2018; Deng 2019), the adverse reaction rate was mentioned in 7 studies (Li et al. 2015; Zhang 2015; Fan 2017; Ying and Shi 2017; Zeng et al. 2017; Hang 2018; Peng 2019), 2 of them recorded serum total IgE levels (Ying and Shi 2017; Zeng et al. 2017) and 7 articles described immune indices (Li et al. 2015; Sun 2016; Qian 2017; Ying and Shi 2017; Zeng et al. 2017; Sheng et al. 2018; Deng 2019).

#### Total response rate

**Figure 4.**
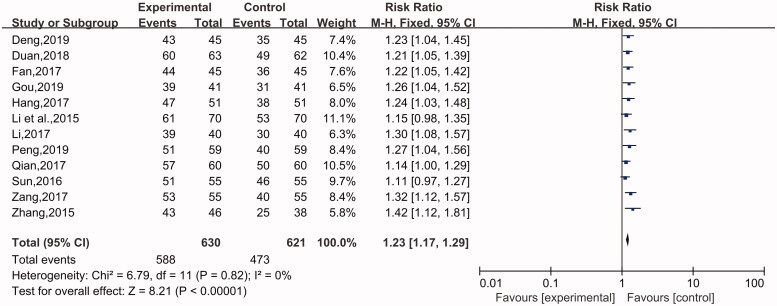
Total response rate.

This indicator was used in 12 studies (Li et al. 2015; Zhang 2015; Sun 2016; Fan 2017; Qian 2017; Ying and Shi 2017; Zang 2017; Duan 2018; Hang 2018; Deng 2019; Gou 2019; Peng 2019), involving 1251 cases in total, including 630 in the experimental group and 621 in the control group. A fixed-effect model was applied for analysis because there was no heterogeneity (*p* = 0.82, I^2^ = 0%). There was a difference between the experimental group and the control group. The patients treated with the combination of desloratadine and compound glycyrrhizin showed a higher response rate than the patients treated with desloratadine alone (*n* = 12, RR = 1.23, 95% CI: 1.17 to 1.29, Z = 8.21, *p* < 0.00001) (Figure 4).

#### Cure rate

**Figure 5.**
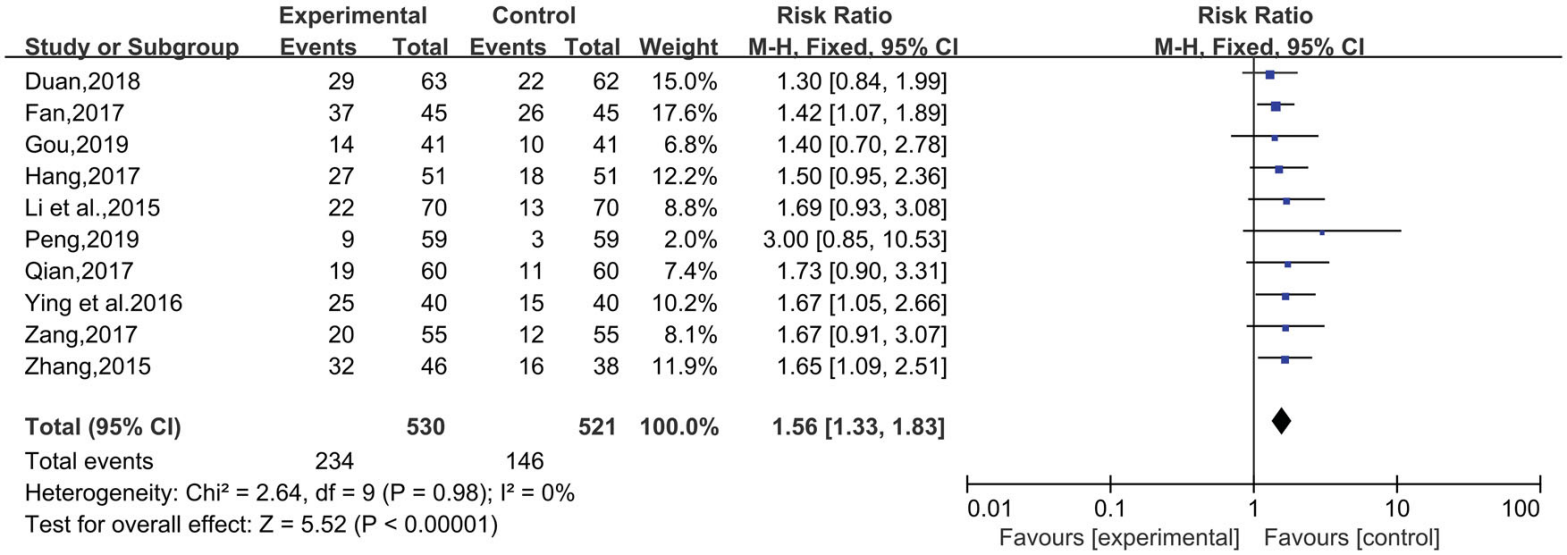
Cure rate.

This indicator was recorded in 11 studies (Li et al. 2015; Zhang 2015; Fan 2017; Qian 2017; Ying and Shi 2017; Zang 2017; Zeng et al. 2017; Duan 2018; Hang 2018; Gou 2019; Peng 2019), involving 1231 cases in total containing 620 in the experimental group and 611 in the control group. A fixed-effect model was applied for analysis because of no heterogeneity (*p* = 0.96, I^2^ = 0%). A significant difference was shown between the experimental group and the control group. The patients treated with the combination of desloratadine and compound glycyrrhizin showed a higher cure rate than the patients treated with desloratadine alone (*n* = 11, RR = 1.50, 95% CI: 1.30 to 1.73, Z = 5.64, *p* < 0.00001) (Figure 5).

#### Recurrence rate

**Figure 6.**
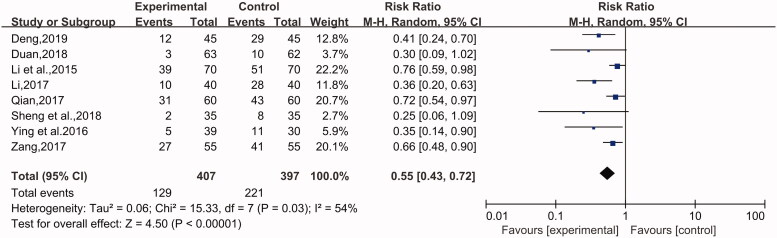
Recurrence rate.

This indicator was used in 7 studies (Li et al. 2015; Qian 2017; Ying and Shi 2017; Zang 2017; Duan 2018; Sheng et al. 2018; Deng 2019), involving 714 cases with 367 in the experimental group and 357 in the control group. A fixed-effect model was applied for analysis due to nonobvious heterogeneity (*p* = 0.10, I^2^ = 44%). There was a significant difference indicated between the experimental group and the control group by analysing the related data. The patients treated with the combination of desloratadine and compound glycyrrhizin showed a lower recurrence rate than the patients treated with desloratadine alone (*n* = 7, RR = 0.61, 95% CI: 0.52 to 0.71, Z = 6.16, *p* < 0.00001) (Figure 6).

#### Rate of adverse reactions

**Figure 7.**
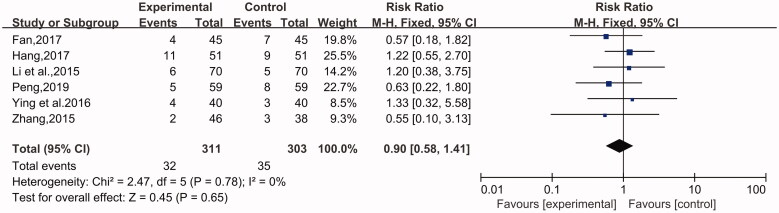
Rates of adverse reactions.

Among 14 RCTs, 7 studies evaluated the rate of adverse reactions (Li et al. 2015; Zhang 2015; Fan 2017; Ying and Shi 2017; Zeng et al. 2017; Hang 2018; Peng 2019), comprising 794 incident cases of CU, including 401 in the experimental group and 393 in the control group. No heterogeneity was discovered in this analysis. In addition, the analysis data indicated that the incidence of adverse reactions was slightly lower in the experimental group than in the control group. Furthermore, there was no statistical significance in the analysis results (*n* = 7, RR = 0.88, 95% CI: 0.58 to 1.31, Z = 0.64, *p* = 0.52) (Figure 7).

#### Immune system function

##### Serum total IgE level

**Figure 8.**
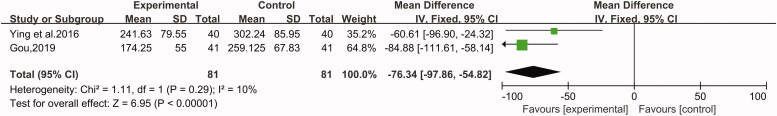
Serum total IgE level.

Only 2 studies (Ying and Shi 2017; Zeng et al. 2017) including 260 patients measured this indicator, with 130 cases per group. A fixed-effect model was employed for this analysis because there was no heterogeneity (P = 1.00, I^2^ = 0%). According to this model, a significant discrepancy was found between the two groups. After their respective treatments, the participants in the experimental group showed an obviously lower level of IgE than those in the control group (*n* = 2, MD = 60.62, 95% CI: −80.67 to −40.56, Z = 5.92, *p* < 0.00001) (Figure 8).

##### The level of CD4+ T and CD8+ T cells

**Figure 9.**
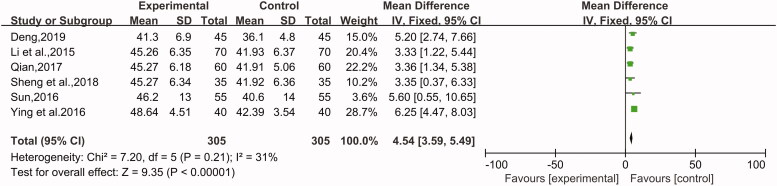
The level of CD4+ T cells.

**Figure 10.**
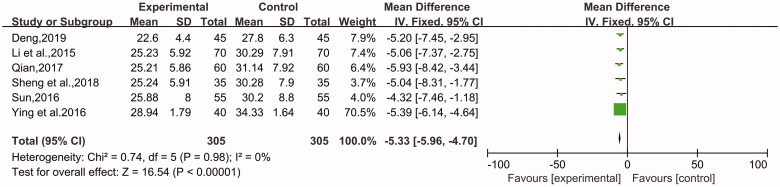
The level of CD8+ T cells.

The data of these two indicators that affect the immune system were recorded in 7 studies (Li et al. 2015; Sun 2016; Qian 2017; Ying and Shi 2017; Zeng et al. 2017; Sheng et al. 2018; Deng 2019) with a total of 790 patients, including 395 patients in the experimental group and 395 patients in the control group. One was CD4+ (MD = 4.88, 95% CI: 3.73 to 6.04, Z = 8.27, *p* < 0.00001) (Figure 9), and the other was CD8+ (MD = −5.35, 95% CI: −5.88 to −4.8, Z = 19.54, *p* < 0.00001) (Figure 10). In addition, a random-effect model was used to describe the CD4+ T cells due to high heterogeneity (*p* = 0.06, I^2^ = 50%), while the CD8+ T cells were subjected to a fixed-effect model for analysis (*p* = 0.99, I^2^ = 0%). The patients with CU who received combination therapy had a significantly more apparent improvement in the function of the immune system compared with the control group.

### Publication bias

**Figure 11.**
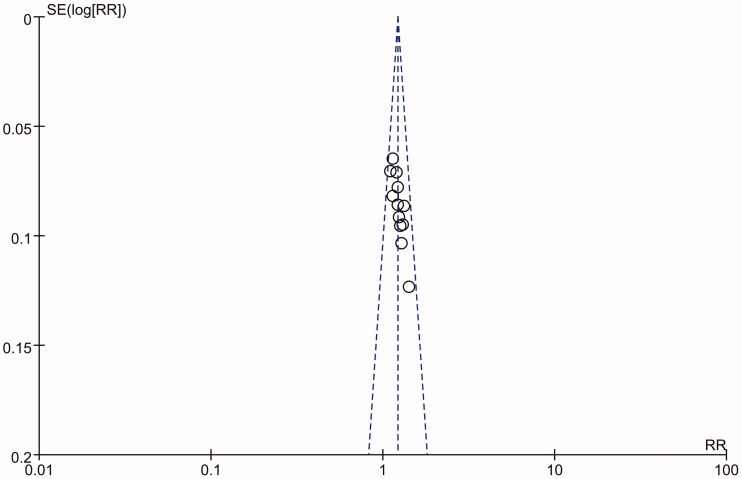
The funnel plot of the publication bias.

Publication bias was evaluated with a funnel plot of all 12 included studies. The 12 studies (Li et al. 2015; Zhang 2015; Sun 2016; Fan 2017; Qian 2017; Ying and Shi 2017; Zang 2017; Duan 2018; Hang 2018; Deng 2019; Gou 2019; Peng 2019) were distributed on both sides of the funnel plot, but there was some asymmetry in the funnel plot, indicating that the results obtained from the included literature may have publication bias. The funnel plot of the total response rate is shown in Figure 11.

## Discussion

### Why we undertook this analysis

CU is one of the most common diseases in dermatology with the clinical characteristics of irritability, multiple, recurrent, prolonged and non-healing. In clinical work, the causes of CU are complex and difficult to detect, and its impact on the quality of life of the patients is quite serious (Hu 2016; Wang and Xu 2017). Therefore, it is urgent to find an ideal method to control and treat it. The first-line treatment for CU is applying antihistamines to relieve the symptoms (Lai et al. 2010). Although the therapeutic effect is acceptable, due to the complexity of the pathogenesis of CU, the control of recurrences with a single drug is not ideal, and the long-term efficacy also needs to be improved (Gou 2019; Bao et al. 2020). Among the different pathogeneses, autoimmunity is considered to be one of the most important causes of CU. As a consequence, it has been reported that antihistamines combined with immune modulators in the treatment of CU can achieve a better therapeutic effect and a lower recurrence rate (Chen et al. 2017; Wu 2017).

Type I allergic urticaria is most common in clinical work. Serum IgE, as one of the important indicators reflecting the degree of allergic reaction in the body, plays a key role in mutational reactive diseases (Zhu et al. 2017). Meanwhile, IgE may be related to CD4+ T and CD8+ T cell levels in the body. Previous reports have suggested that altering the proportions of T lymphocyte subsets can lead to CU (Li et al. 2010). Another study also confirmed that CD4+ T cells play a major role in antigen recognition in the CU. The decrease in CD4+ T cells and the increase in CD8+ T cells may lead to CU (Zhu et al. 2021). According to its possible pathogenesis and the current treatment recommendations, our group chose to assess the efficacy of desloratadine in combination with compound glycyrrhizin in treating CU.

A study published in 2019 showed that desloratadine had better efficacy in the treatment of urticaria than other antihistamines, and its safety was reliable (Lang et al. 2019). The main components of glycyrrhizin are glycyrrhizin and glycine, which have ideal anti-inflammatory and immune mediation effects (Wang and Li 2013). Although long-term use of glycyrrhizin can cause electrolyte metabolism abnormalities, glycyrrhizinate containing glycine can be combined with l-cysteine hydrochloride to inhibit or reduce electrolyte metabolism disorders (Grob et al. 2005). In addition, compound glycyrrhizin can improve the level of CD4+ T cells, reduce the level of CD8+ T cells, inhibit the release of arachidonic acid, and reduce leukotrienes (Wang 2016). Desloratadine in combination with compound glycyrrhizin can produce effects on different aspects of this disease, forming synergetic and complementary advantages in immune regulation and antiallergy, prolong the time of drug action, reduce the sensitivity of the body, enhance the clinical efficacy and reduce the recurrence of the disease. Furthermore, compound glycyrrhizin is also a kind of Chinese medicine compound preparation (Lin et al. 2014), and combined with desloratadine, glycyrrhizin meets the standard of integrated Chinese and Western medicine treatment.

In this study, we systematically evaluated the functions of desloratadine combined with compound glycyrrhizin in treating CU. Through a meta-analysis of the published studies, this research increased the sample size, enhanced the research credibility and provided reliable data support for its clinical use. In addition, we expect to verify that a combination of antihistamines and immune modulators can enhance the therapeutic effect on patients with CU compared with using antihistamines alone based on objective evidence.

### What we learned from this analysis

First, the results for the total response rate and cure rate suggested that the therapeutic effect of desloratadine in combination with compound glycyrrhizin in the treatment of patients with CU was higher than that of desloratadine alone. In particular, the outcomes for the cure rate indicated superiority. A better prognosis was shown through reductions in the recurrence rate with this combination therapy.

Second, the results for the adverse reaction rate revealed that there was no statistically significant difference between desloratadine combined with compound glycyrrhizin and individual use of desloratadine. In addition, both of them showed a low rate of adverse reactions. Thus, the combination with compound glycyrrhizin did not increase the incidence of adverse reactions and the combination therapy was relatively safe.

Third, the outcomes of the immune indicators showed that a combination of the two drugs can achieve a better effect in increasing CD4+ T cells and decreasing the levels of IgE and CD8+ T cells than a single use of desloratadine. The balance of CD4+ T and CD8+ T cells was improved, while the level of IgE was significantly reduced in the experimental groups. The compound glycyrrhizin has good regulation of immune function, which can strengthen the immune regulatory function of the body to better balance CD4+ T and CD8+ T cells and reduce IgE. It was also hypothesised that the application of compound glycyrrhizin might enhance the antihistamine action of desloratadine to cause the IgE levels to be significantly lower and thus control any recurrence (Li et al. 2010).

Finally, the results of this study almost satisfied our goal of this meta-analysis. In addition, our conjecture can be confirmed to some extent. However, because some clear and unclear limitations remain in our analysis, the results of our analysis are not complete, perfect and reliable, and deeper analysis is required in the future.

### What we plan to do based on this analysis

Our team plans to select more relevant studies. Additionally, further meta-analysis will be performed on other experimental indicators that are not included in this study. In addition, we still intend to analyse the effect of other antihistamines commonly used in the clinic, such as ebastine, levocetirizine and mizolastine, combined with compound glycyrrhizin or other drugs that can be used as immune modulators in treating CU to observe and verify the clinical advantages of antihistamines combined with immune modulators. Also, we plan to analyse and compare the effectiveness of each combination from the perspective of bioinformatics and the population tendency to provide more recommendations for clinical use. Finally, we found that some decoctions applying glycyrrhiza as their sovereign drug can also be used to treat CU (Zhang et al. 2021), so we intend to analyse the efficacy of this treatment and compare it with the application of compound glycyrrhizin in treating CU. However, relevant studies are seldom now. We hope that more attention could be put on this field to help support our further analysis.

## Limitations

The curative effect of desloratadine in combination with compound glycyrrhizin in treating patients with CU was assessed for the first time by applying the Methods for Cochrane Systematic Review in this analysis. In addition, we also analysed some immune indices that are associated with CU to demonstrate an improvement of the patients’ immune function. However, due to the limited number of cases and the low quality of the articles we included, this study fails to present a reliable and compelling verification result. The limitations of this study are as follows: 1) the course of the disease and the age range of the patients among the included articles had some differences, which may result in differences in treatment efficiency; 2) the doses of the drugs were different among the experimental groups in 14 articles and this may have an impact on the results of this research. To be specific, different doses of desloratadine may lead to different degrees of adverse reactions for individuals, and high doses of compound glycyrrhizin are more likely to induce pseudoaldosteronism (Gao et al. 2016). Different doses of compound glycyrrhizin may also influence the effect of this combine treatment in reducing side effects; 3) the articles were all from Chinese databases and the patients in all of the articles we included were Chinese, which is very likely to cause ethnic and geographical biases; 4) we limited the language to English and Chinese when searching the literatures which may lead to linguistic deviations; and 5) the included articles had a low quality on average. Although all studies adopted random experiments, only 6 of them explained the specific method of random grouping, and few of them mentioned the blinding method as well as allocation concealment.

All of these limitations may increase information bias and selection bias in this analysis and lead to some heterogeneity. To address these limitations, we will expand the retrieval area and collect additional data in different countries and regions to conduct a more comprehensive analysis in the future. However, there are few randomised controlled studies about the combination of desloratadine and compound glycyrrhizin in treating CU. In addition, few RCTs have focussed on the treatment of CU with immune modulators. We hope that more high-quality, specific and large RCTs with long-term follow-up will be performed in the future so that this promising combination therapy can be better confirmed and explained.

## Conclusion

The analysis results indicated that the therapeutic effect, including the cure rate and total response rate, of patients treated with desloratadine in combination with compound glycyrrhizin was more beneficial than when using desloratadine alone. The recurrence rate was lower, and the immune function was improved, while the safety of both intervention measures was similar. According to the results of this meta-analysis and the present theoretical basis, the combination of desloratadine and glycyrrhizin can be a promising treatment for CU. In addition, the superiority and advantages of this combination medication in treating CU was demonstrated with the clinical data in this study. However, due to the low quality of the included articles and limitations of the drug selection in this study, higher-quality analysis needs to be performed in the future.
